# High shocking and pacing impedances due to defibrillation lead calcification

**DOI:** 10.1007/s10840-019-00685-x

**Published:** 2019-12-18

**Authors:** Robert G. Hauser, Jay Sengupta, Susan Casey, Chuen Tang, Larissa I. Stanberry, Raed Abdelhadi

**Affiliations:** 1 Minneapolis Heart Institute Foundation, 920 East 28th Street, Minneapolis, MN 55407 USA; 2 Long Lake, USA

**Keywords:** Impedance, Defibrillation lead, Calcification, Malfunction

## Abstract

**Purpose:**

We have reported the calcification of Endotak defibrillation leads that required replacement. The aim of this study was to assess calcified Endotak Reliance leads in the Food and Drug Administration Manufacturer and User Facility Device Experience (MAUDE) database and compare them to calcified Sprint Fidelis, Sprint Quattro Secure, Riata, and Durata leads in MAUDE.

**Methods:**

We searched the MAUDE database from 2008 to 2019 for defibrillation lead calcification using the terms “calcium,” “calcification,” and “calcified”. Included were explanted leads whose manufacturers found calcium on the shocking and/or pacing electrode.

**Results:**

The MAUDE search identified 113 calcified defibrillation leads that qualified for the study, including 109 Endotak Reliance leads, 1 Sprint Quattro Secure lead, 2 Durata leads, 1 Riata ST lead, and no Sprint Fidelis lead. The sign of calcification was a gradual increase in shocking or pacing impedance. Average implant time was 7.4 ± 3.1 (range: 1.3–16.5) years. Only Endotak Reliance leads had shocking coil calcification (*n* = 72; 66.0%) and five (6.9%) of these failed defibrillation threshold (DFT) testing. Distal pacing electrode calcification affected 55 (50.4%) Endotak Reliance leads. The four other leads had pacing ring electrode calcification only.

**Conclusion:**

Endotak Reliance defibrillation leads appear prone to shocking coil and/or distal pacing electrode calcification. High impedances may compromise defibrillation and pacing therapy. Patients who have these leads should be monitored; those exhibiting high shocking impedances should be considered for DFT testing. Lead replacement should be considered for pacemaker-dependent patients whose leads exhibit progressively high impedances.

## Introduction

Calcification of tissue surrounding a defibrillation lead may complicate extraction, but calcification of a shocking coil or pacing electrode may increase impedance, limit current flow, and potentially compromise effective therapy [1]. Recently, we reported the calcification of Endotak Reliance (Boston Scientific, St. Paul, MN) high-voltage (HV) defibrillation leads that required replacement [2, 3].

The aim of this study was to assess all clinical events associated with confirmed Endotak Reliance lead calcification as described by the manufacturer in reports publicly available in the Food and Drug Administration’s (FDA) Manufacturers and User Facility Device Experience (MAUDE) database. These results were compared to similar data for Sprint Fidelis and Sprint Quattro Secure (Medtronic Inc., Minneapolis, MN) and Riata and Durata (Abbott/St. Jude Medical, Sylmar, CA) HV defibrillation leads.

## Methods

### Study design

This is a retrospective study of Endotak Reliance leads that were removed from patients, returned to Boston Scientific for analysis, and found to have calcification of one or two shocking coils and/or the distal pacing electrode. The results are compared to four HV defibrillator lead models from two other manufacturers.

### Leads

*Endotak Reliance* [4] is a multilumen silicone steroid-eluting HV defibrillator lead that has one or two bifilar platinum clad tantalum shocking coil electrodes. The pacing electrodes are integrated, whereby the distal shocking electrode serves as the anode, and the distal cathodal electrode is composed of iridium oxide-coated titanium. The shocking coils of Reliance G and 4-SITE models are covered with Gore-expanded polytetrafluoroethylene (ePTFE) that prevents tissue ingrowth around and in between the coil filars. Normal HV shocking impedance is 20–125 Ω. Normal pacing impedance is 450–1800 Ω for passive fixation leads and 300–1200 Ω for active fixations leads, except for 4-Front models that are 300–1200 Ω for both active and passive fixation leads. Since the first implant in 2000, approximately 950,000 Endotak Reliance leads have been distributed worldwide.

*Sprint Fidelis* and *Sprint Quattro Secure* are multilumen silicone steroid-eluting HV defibrillator leads that have one or two ethylene tetrafluoroethylene (ETFE)-coated shocking coil electrodes consisting of 19 platinum-clad tantalum filaments. The platinized platinum alloy electrodes are true bipolar with the distal active or passive tip as the cathode and proximal ring electrode as the anode. Normal shocking impedance is typically 20–100 Ω, and the normal pacing impedance is typically 300 to 1000 Ω. Since their introductions in 2001 and 2004, approximately 720,000 Sprint Quattro Secure and 205,000 Sprint Fidelis leads have been implanted in the USA.

*Riata* and *Durata* are multilumen silicone steroid-eluting HV defibrillator leads with redundant conductors and one or two platinum-iridium alloy-shocking coils. The titanium nitride-coated platinum-iridium alloy electrodes are true bipolar with the distal active or passive tip as the cathode and proximal ring electrode as the anode. Normal shocking impedance is typically 20–100 Ω, and the normal pacing impedance is usually 300–1000 Ω. Since their introduction in 2002 and 2009, approximately 151,000 Riata and 311,000 Durata leads have been implanted in the USA.

### FDA MAUDE database

The MAUDE database contains reports of adverse events involving medical devices that are reported to US manufacturers by users worldwide. Included are medical devices that are or remain implanted, or have been explanted, and medical devices that are used externally. MAUDE medical device reports (MDR) are publicly available online for the previous 10 years at www.fda.gov/cdrh/ maude.html. Manufacturers are required to report the results of their analyses of returned devices.

During August 2019, the MAUDE database was queried for Endotak Reliance leads using the simple search terms “calcium,” “calcification,” and “calcified” for the years 2008 through May 2019. Identical searches were made for Sprint Fidelis and Sprint Quattro and Riata and Durata leads. The following data were extracted from the reports identified by our search: (1) dates of manufacture, clinical event, when the explanted device was received by the manufacturer; (2) clinical information and signs of device failure that were reported to the manufacturer; and (3) results of the manufacturer’s analysis of the returned lead.

Implant times were estimated using the date of manufacture, and the date the lead was returned to the manufacturer; as reported previously [3], 4.7 months were subtracted to adjust for shipping.

### Study population

A lead was included in the study if (1) it was implanted, removed, and returned to the manufacturer for analysis and (2) if the manufacturer concluded that the one or two coils and/pacing electrodes were calcified or had evidence of calcium or calcification. Excluded were leads that had clinical or electrical signs of calcification but were not removed or analyzed by the manufacturer.

### Statistics

Variables were summarized using counts (%) for categorical variables and mean ± standard deviations or medians (interquartile ranges) for continuous variables, as appropriate. The differences in median implant times between different calcification locations and between Gore-covered and non-covered leads were estimated using quartile regression. The resulting estimates and the corresponding 95% confidence intervals are reported. The statistical analysis was performed using R v 3.6.1 (R Foundation for Statistical Computing, Austria) in R studio v 1.1.463 (R Studio, Inc.) with quantreg package.

## Results

The MAUDE search identified 113 calcified HV defibrillation leads that qualified for the study, including 109 Endotak Reliance leads (96.5%), 1 Sprint Quattro Secure lead, 2 Durata leads, 1 Riata ST lead, and no Sprint Fidelis lead.

Table 1 provides the electrical signs and locations of calcification. A gradual rise in impedance was the cardinal sign of shocking coil and distal pacing electrode calcification. Only Endotak Reliance leads exhibited shocking coil calcification, resulting in high out-of-range (OOR) shocking impedances (> 125 Ω), and five cases of failed defibrillation threshold (DFT) testing.

**Table 1:** Signs of lead failure and location of calcium on shocking coils and pacing electrodes

	Endotak Reliance	Sprint Quattro Secure	Durata & Riata
No. Leads	109	1	3
Sign			
High OOR* shocking impedance Failed DFT test	67 4	–	–
High OOR pacing impedance Loss of capture Increased pacing threshold	38 4 18	1 - 1	3 - 2
High OOR shocking & pacing impedance Failed DFT Increased pacing threshold	3 1 1	–	–
Dislodgement	1	–	–
Calcium locations			
Proximal shocking coil only	4	–	–
Distal shocking coil only Failed DFT test	25 2	–	–
Proximal and distal shocking coils Failed DFT test	25 2	–	–
Pacing electrode only	37	1	3
Distal shocking coil and pacing electrode Failed DFT test	9 1	–	–
Proximal shocking coil and pacing electrode	2	–	–
Both shocking coils and pacing electrode	7	–	–

*OOR, out of range; DFT, defibrillation threshold

Isolated high OOR pacing impedance, usually > 2000 Ω, was the sign of failure for 38 Endotak Reliance and the other 4 leads; increased pacing threshold was common, but loss of capture was infrequent, and none resulted in a serious adverse event. One Endotak Reliance chronic lead dislodged, and calcified tissue was found in the helix.

**Fig. 1:**
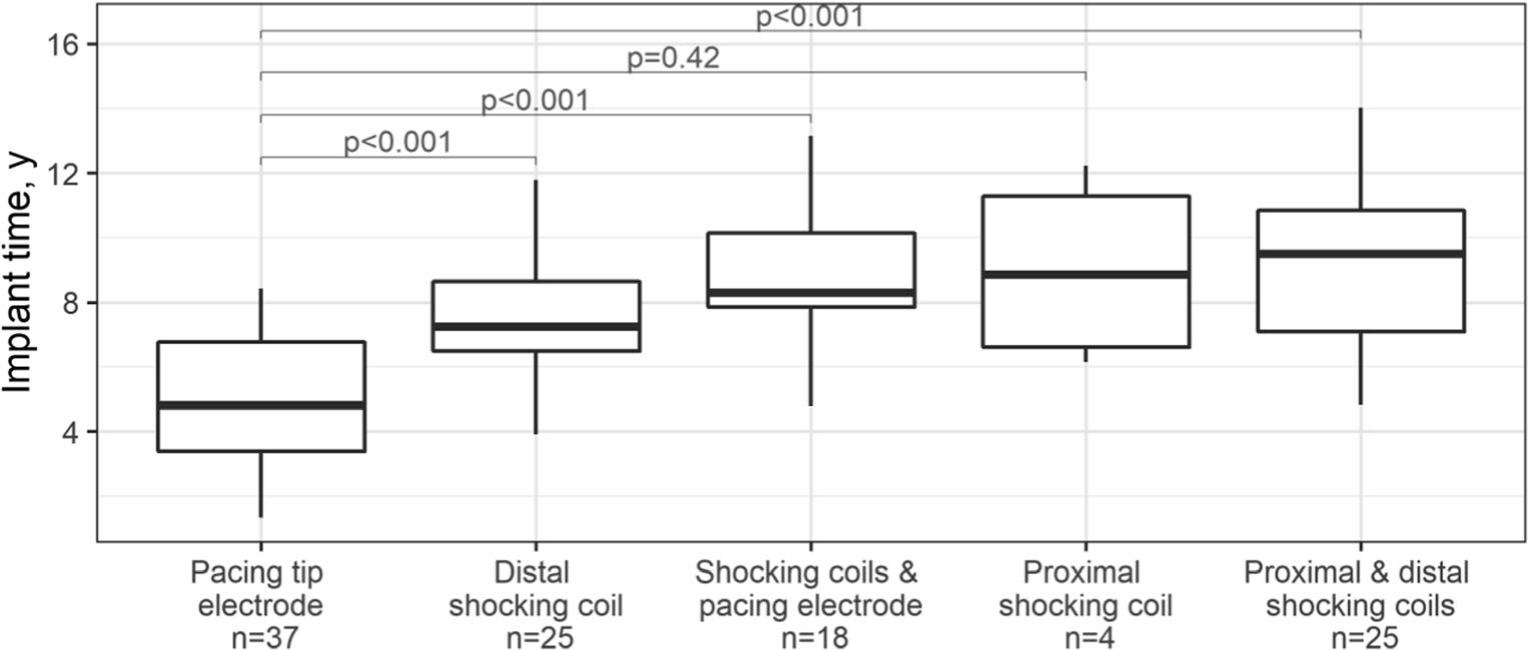
Box graph of Endotak Reliance defibrillation lead implant times according to the location of calcification

Overall, the average implant time for calcified Endotak Reliance leads was 7.4 ± 3.1 (range: 1.3–16.5) years. Figure 1 shows the median Endotak Reliance lead implant times according to the location of calcification. Pacing electrode calcification appeared significantly earlier than calcification of the distal shocking coil, both shocking coils and the distal pacing electrode (*p* < 0.001).

Calcification of the four Durata, Riata ST, and Sprint Quattro Secure leads was confined to the ring electrode (anode) and occurred an average of 5.7 ± 1.4 (range: 3.8–7.1) years after implant.

The majority of calcified Endotak Reliance leads had Gore ePTFE-covered shocking coils (Table 2). The median implant times for Gore covered and non-Gore covered leads were not significantly different (*p* = 0.36). Gore-covered leads were the only leads with calcification confined to the shocking coils. All non-Gore covered leads had high pacing impedances and distal pacing electrode calcification; three of these non-Gore leads also had calcium on a shocking coil, but none had a high shocking impedance.

**Table 2:** Endotak Reliance calcification with and without Gore ePTFE-covered shocking coils

	With Gore	Without Gore
Number	102	7
Median implant time (IQR), yrs	10.4 (4.3, 11.7)	7.3 (5.5, 8.9)*
Sign of Failure		
High shocking impedance	66	-
High pacing impedance	31	7
High shocking and pacing impedance	3	-
Location of calcification		
Proximal shocking coil	4	-
Distal shocking coil	25	-
Pacing electrode	33	4
Both shocking coils	25	-
One or both coils & pacing electrode	14	3

*p = 0.36

IQR = interquartile ranges

Table 3 provides information and data from MAUDE for calcified Endotak Reliance leads. Included are the five leads (#1–5) that failed defibrillation threshold testing and the lead (#6) with a calcified distal tip electrode that was associated with death during extraction. Also included are representative examples of clinical findings that are correlated with the results of the manufacturer’s analyses (#7–12). Code 1005 was seen after shock tests in cases 1, 7, and 8. This code is displayed on the Boston Scientific Programmer when a shock may not have delivered full therapy which is caused by an open-circuit condition or when a high out-of- range impedance is encountered; according to the manufacturer, this may be caused by calcification or fracture.

**Table 3:** The manufacturer’s description of clinical events, results of returned lead analyses, and other pertinent findings related to Endotak Reliance calcification

#	MDR #	Endotak Model #	Implant (years)	Sign/Reason for Failure Outcome	Manufacturer’s Description
1	8,421,797	Reliance G 185	14.0	High OOR shocking impedance > 125 Ω after HV shock Code 1005 after shock* Failed DFT test requiring external rescue Lead replaced	“Calcified bodily fluid was observed on the distal and proximal shocking coils. This could have contributed to the observed high shock impedance measurements.”
2	5,372,653	Reliance 184	11.8	High OOR shocking impedance > 125 Ω Failed DFT test Lead replaced	“Visual observation noted a build-up of calcification on the lead shocking electrodes that created a non-conductive barrier between the lead’s shocking electrodes and the patient’s heart tissue. Laboratory analysis confirmed the reported observations and concluded that the calcification was the root cause.”
3	5,598,111	Reliance SG 181	8.6	High OOR shocking impedance > 125 Ω Failed DFT test Lead replaced	“Only the distal segment of the lead was returned… Visual inspection on the returned segment of lead showed calcium noted on the distal shocking coil. Depending on how much calcification was on the lead before the lead was explanted, the impedance measurement could have been affected.”
4	6,024,239	Reliance 4-SITE 292	4.8	High OOR shocking impedance > 125 Ω Failed DFT test Lead replaced	“Laboratory analysis concluded that the high out of range shock impedance measurements were likely due to the calcification on the distal spring electrode, creating a non-conductive barrier between the lead’s shocking coil and tissue, thereby gradually increasing the impedance seen by the device over time as the calcification builds up.”
5	3,948,035	Reliance G 184	8.5	High OOR shocking impedance > 125 Ω Failed DFT test Lead replaced	“Visual inspection revealed calcified body fluid covering the distal and proximal shocking coil extending to the distal tip of the lead. The impedance could have been affected depending on how thick the calcification was. In conclusion, the allegation of high shock impedance measurement was confirmed.”
6	7,099,566	Reliance G 175	7.2	High OOR pacing impedance > 3000 Ω Death during lead extraction	“Calcified body fluid and possible tissue fully encapsulated the lead tip…The lead passed electrical testing. The pacing impedance measurement could have been affected, depending on how much calcification was on the lead tip while the lead was implanted.”
7	3,041,259	Reliance SG 180	8.6	High OOR shocking impedance Code 1005 after command shock Lead replaced	“…calcification has built up on the lead’s distal coil creating a non-conductive barrier as the calcification built up. When the calcification built up, a gradual increase in impedance was seen by the device over time. The high impedance was confirmed by analysis due to calcification which has built up on the leads distal coil.”
8	4,957,633	Reliance SG 180	4.0	Gradually increasing shock impedances → 1.1 J shock normal impedance → 41 J shock Code 1005 Lead replaced	“…calcification along with tissue [was]noted on the lead body insulation and on most of the distal spring electrode…X-ray did not reveal any conductor fractures…calcification may have accounted for the observation of high shock impedance measurements. Build-up of calcification on the lead’s shocking coil can create a non-conductive barrier between the lead’s shocking coil and the patient’s heart tissue, thereby gradually increasing the impedance seen by the device over time.”
9	3,586,847	Reliance G 185	6.8	High OOR shocking impedance Lead replaced	“…there was calcification that encapsulated the entire proximal coil…Both the rate/sense and distal HV conductors were found to be electrically continuous; however, initial resistance testing on the proximal HV conductor did not meet specifications…After approximately 10% of the calcification was removed from the proximal coil the lead exhibited normal measurements…Analysis confirmed that the OOR impedance measurements were the result of a calcification layer that encapsulated the entire proximal shocking coil.”
10	7,658,347	Reliance G 171	3.6	High OOR pacing impedance > 3000 Ω High pacing threshold (7 V @ 2 msec) Lead replaced	“Calcified bodily fluid was noted on the lead tip and lead tip mesh screen…x-ray examination and continuity testing revealed no conductors fracture on returned segment of lead. Visual inspection showed calcification on distal tip of lead. Impedance measurements may have been affected depending on the extent of the calcification on the lead tip and lead tip mesh screen prior to lead explant.”
11	2,778,405	Reliance G 185	7.9	Remote monitoring detected OOR impedance Clinic test reveals all shock vectors > 125 Ω Lead replaced	“Visual inspection revealed evidence of calcification over the distal and proximal shocking coils. Depending on how much calcification was on the tip before the lead was explanted, the impedance measurement could have been affected. Testing on a passive fix mesh tip lead did shown that as the calcified material is removed, the impedance drops. Analysis confirmed the clinical observations.” ‘
12	5,174,431	Reliance G 185	10.1	High OOR shocking impedance Testing revealed normal impedance when the proximal coil was excluded Follow-up testing revealed OOR shocking impedance Lead replaced	“Visual inspection noted calcification build-up on the proximal and distal spring electrodes. Resistance tests were completed to assess lead electrical performance. Measurements throughout these tests were within normal limits. Although the returned segments were electrically continuous, calcification on the proximal and distal electrodes could cause an increase in impedance measurements over time.”

MDR, medical device reporting number; OOR, out-of-range; HV, high voltage; DFT, defibrillation threshold; msec, millisecond

*Code 1005 indicates failure to deliver therapy due to open circuit conditions and, according to manufacturer, may be caused by calcification, lead fracture, or under-inserted pin in the connector

## Discussion

The results of this study suggest that Endotak Reliance defibrillation leads are prone to shocking coil and distal pacing electrode calcification that may result in high impedances and potential failure to defibrillate or pace. To our knowledge, this is the first study to report high defibrillation thresholds and inability to defibrillate due to shocking coil calcification. A second unique finding is the unusually high number of Endotak Reliance leads with calcified distal pacing electrodes. This study found only four HV defibrillation leads from other manufacturers that had pacing electrode calcification, and none had calcified shocking coils.

The incidence of Endotak Reliance calcification is unknown. Medical device reporting in the USA relies on a passive surveillance system that suffers from under-reporting, and MAUDE does not provide data for the actual number of devices at risk. However, given the large volume of Endotak Reliance leads implanted, it is likely that malfunction due to coil or electrode calcification is infrequent. Patients who have chronic kidney disease or hypercalcemia may be more susceptible to lead-electrode calcification [5].

The average implant time for the leads in this study was 7.4 years, but the range was wide (1.3 to 16.5 years). Distal pacing electrodes appeared to calcify earlier than shocking coils, and longer implant times were associated with more extensive calcification. Calcification is a progressive process, and its hallmark is a gradual increase in shocking and/or pacing impedance. The information in MAUDE was insufficient to characterize the time course of calcification or the rate of rise of impedances to critical values.

As is true of any lead, physicians following patients who have Endotak Reliance leads should be especially alert to a progressive increase in impedance and, when observed, should institute heightened surveillance, including remote monitoring. Impedance values will vary according to the measurement methods employed. Painless, subthreshold, low-voltage pulses are commonly used to measure lead impedances in HV leads. These low-voltage impedances (LVZ) appear to correlate with high-voltage impedances in normal leads [6], and they may reliably detect HV conductor fractures [7]. However, recently Swerdlow et al. [8] found that LVZ methods may be insensitive to lead-housing insulation breaches. Impedance measurements obtained by LVZ have not been studied in calcified leads, and thus, we do not know if LVZ-derived impedances reliably reflect the true impedance. High shocking impedance may alter the defibrillation waveform and potentially diminish delivered energy. For these reasons, we suggest that formal DFT testing be performed when high shocking impedances characteristic of calcification are encountered. Leads that fail DFT testing should be replaced. Similarly, leads with high pacing impedances and thresholds in pacemaker-dependent patients could be considered for replacement.

The reason(s) for Endotak Reliance calcification is speculative. Shocking coil calcification may be related to the presence of Gore ePTFE, which has occasionally calcified in other cardiovascular applications [9, 10]. However, some calcium was found on a shocking coil of four of the seven non-Gore Endotak Reliance leads in this study, but none of these had a high shocking impedance. The distal pacing electrode calcification may be caused by a different process and could be related to the integrated electrode configuration, whereby the distal shocking electrode serves as the pacing anode.

The only death in our study occurred during lead extraction. Since lead calcification is a known extraction risk [11, 12], studies are needed to determine the safest approach to managing non-infected Endotak Reliance leads that exhibit high shocking and/or pacing impedances that may be due to calcification.

This study has limitations. We assumed that the fraction of leads reported in MAUDE was the same for the three manufacturers and was independent of the cause for lead removal or facility where it was implanted. According to manufacturers’ product performance reports, only 4–10% of removed leads are returned for analysis. Further, the majority of failed leads is abandoned in situ. Thus, it is very likely that the actual number of leads that have failed due to shocking coil or distal pacing electrode calcification is substantially higher than the 113 leads in this report. The MAUDE data did not allow us to assess the efficacy of painless or HV impedance measurements for identifying high out-of-range impedances. It is possible that a higher proportion of Endotak leads were removed because the Gore covering facilitated extraction. It is also possible that the small number of calcified leads reported by Medtronic and Abbott/St. Jude Medical was due, in part, to differences in analytic techniques.

## Conclusion

For unclear reasons, Endotak Reliance defibrillation leads appear to be prone to shocking coil and/or distal pacing electrode calcification. The resulting high impedances may compromise defibrillation and pacing therapy. Patients, who have these leads, should be monitored. Those exhibiting high shocking impedances typical of calcification should be considered for DFT testing and possible lead replacement. Similarly, lead replacement should be considered for pacemaker-dependent patients whose leads exhibit progressively high impedances.

## References

[1] Swerdlow CD, Kalahasty G, Ellenbogen KA (2016). Implantable cardiac defibrillator lead failure and management. J Am Coll Cardiol.

[2] Hauser RG (2019). Calcification of Endotak ICD leads: clinical significance and need for surveillance. J Interv Card Electrophysiol.

[3] Hauser RG, Sengupta J, Schloss EJ, Stanberry LI, Wananu MK, Abdelhadi R (2019). Internal insulation breaches in an implantable cardioverter-defibrillator lead with redundant conductors. Heart Rhythm.

[4] Nisam S, Reddy S (2015). The story of…a lead. Europace.

[5] Kolodzinska A, Kutarski A, Koperski L, Grabowski M, Malecka B, Opolski G (2012). Differences in encapsulating lead tissue in patients who underwent transvenous lead removal. Europace.

[6] Schuchert A, Winter J, Binner L, Kühl M, Meinertz T, Reliance Investigators (2006). Intraoperative comparison of a subthreshold test pulse with the standard high-energy shock approach for the measurement of defibrillation impedance. J Cardiovasc Electrophysiol.

[7] Koneru JN, Gunderson BD, Sachanandani H, Wohl BN, Kendall KT, Swerdlow CD, Ellenbogen KA (2013). Diagnosis of high-voltage fractures in Sprint fidelis leads. Heart Rhythm.

[8] Swerdlow CD, Porterfield JE, Kottam AG, Kroll MW. Why low-voltage shock impedance measurements fail to reliably detect insulation breaches in transvenous defibrillation leads. Heart Rhythm. 10.1016/j.hrthm.2019.05.021. 10.1016/j.hrthm.2019.05.021 31125671

[9] Farivar RS, Sherman SK, Cohn LH (2009). Late rupture of polytetrafluoroethylene neochordae after mitral valve repair. J Thor Cardiovasc Surg.

[10] Fukunaga S, Tomoeda H, Ueda T, Ryusuke M, Aoyagi S, Kato S (2010). Recurrent mitral regurgitation due to calcified synthetic chordae. Ann Thor Surg.

[11] Love CJ (2007). Lead extraction. Heart Rhythm.

[12] Henrikson CA, Brinker JA (2008). How to prevent, recognize, and manage complications of lead extraction. Part III: Procedural factors. Heart Rhythm.

